# Surgeons’ involvement in COVID-19 treatment: a practice by a regional core hospital in Japan to avoid physician burnout

**DOI:** 10.1186/s12913-023-09042-1

**Published:** 2023-01-12

**Authors:** Yugo Matsui, Siyuan Yao, Takashi Kumode, Keisuke Tanino, Ryosuke Mizuno, Yusuke Ogoshi, Shusaku Honma, Teppei Murakami, Takatsugu Kan, Sanae Nakajima, Takehisa Harada, Koji Oh, Takehiro Nakamura, Hiroki Konishi, Shigeki Arii

**Affiliations:** 1 Department of Surgery, Kobe City Medical Center West Hospital, Kobe, Hyogo Japan; 2 COVID-19 Task Force, Kobe City Medical Center West Hospital, Kobe, Hyogo Japan; 3 Department of General Internal Medicine, Kobe City Medical Center West Hospital, Kobe, Hyogo Japan; 4 Department of Diabetes and Endocrinology, Kobe City Medical Center West Hospital, Kobe, Hyogo Japan

**Keywords:** Burn out, Coronavirus disease 2019, Surgeon reallocation, Task sharing

## Abstract

**Background:**

To prevent task accumulation on certain divisions, our institution developed a unique system of allocating inpatient treatment of COVID-19 patients to doctors who were not specialized in respiratory infections. The objective of this study was to investigate whether surgeons can be involved in the COVID-19 inpatient treatment without negatively affecting patient outcome, and how such involvement can affect the wellbeing of surgeons.

**Methods:**

There were 300 patients diagnosed with COVID-19 and hospitalized from January to June 2021, and 160 of them were treated by the redeployed doctors. They were divided into 3 groups based on the affiliation of the treating doctor. Patient characteristics and outcomes were compared between the groups. In addition, the impact of COVID-19 duty on participating surgeons was investigated from multiple perspectives, and a postduty survey was conducted.

**Results:**

There were 43 patients assigned to the Department of Surgery. There were no differences in the backgrounds and outcomes of patients compared with other groups. The surgeon’s overtime hours were significantly longer during the duty period, despite no change in the number of operations and the complication rate. The questionnaire revealed that there was a certain amount of mental and physical burden from the COVID-19 duty.

**Conclusion:**

Surgeons can take part in inpatient COVID-19 treatment without affecting patient outcome. However, as such duty could negatively affect the surgeons’ physical and mental wellbeing, further effort is needed to maintain the balance of fulfilling individual and institutional needs.

## Background

Severe acute respiratory syndrome coronavirus 2 (SARS-CoV-2), responsible for the development of coronavirus disease 2019 (COVID-19), has led to major changes in the workstyles of health care professionals. Overwork and burnout of medical staff have become a matter of concern [1], and the mental health of healthcare professionals have been reported to have worsened in the pandemic’s second year [2]. Reallocation of resources to treat COVID-19 patients have reduced opportunities for surgical experience, which had a negative impact on the workstyle and well-being of surgeons [3–6]. Task shifting of surgeons has been reported in literature, along with the effect on their wellbeing [7–14]. The treatment of COVID-19 patients requires skillsets that surgeons may not necessarily have proficiency in, and thus providing support to their involvement is critical. Yet, the necessary components to such support remain unclear.

**Fig. 1:**
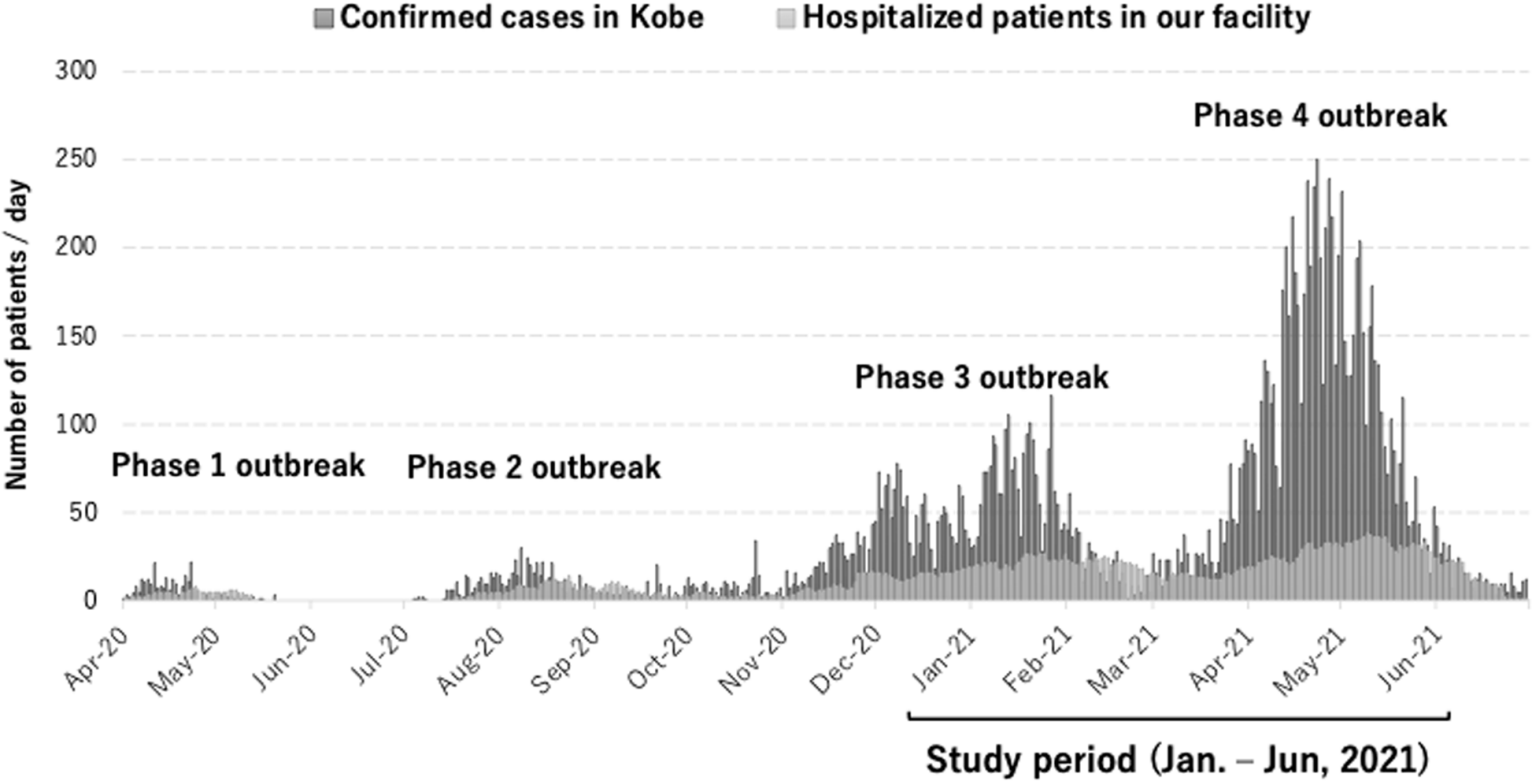
COVID-19-positive cases in Kobe City from April 1, 2020, to June 30, 2021

Japan has experienced 4 major outbreaks by June 2021, with the first case confirmed in January 2020 and the total number of confirmed cases reaching almost 1.6 million as of July 1, 2021 [15]. Kobe City, which is the 5th largest city in Japan with a population of 1.5 million, has also faced 4 outbreaks with over 15,000 confirmed cases [16]. Despite its size, Kobe City has only 2 tertiary care hospitals. Kobe City Medical Center West Hospital, located in an urban area, is a public secondary emergency care hospital with 358 beds. The hospital admitted 487 COVID-19 patients from April 2020 to June 2021, with the highest number of inpatients reaching 38 per day. The transition in the infection situation in Kobe City and the rates of hospitalizations are shown in Fig. 1 [16]. Although we had a maximum capacity of 38 cohorted-ward beds for COVID-19 patients, we could not devote any of our 7 intensive care unit beds to COVID-19 patients since they could not be separated from those for non-COVID-19 patients. Moreover, there were only 2 mechanical ventilators available for COVID-19 use. Therefore, inpatient care was limited to patients with mild to severe illness. Critically ill patients requiring intensive care were transferred to one of the two tertiary hospitals (advanced care facility). As a note, hospital activity (hospitalization, operation volume and beds for non-COVID patients) was cut by 40% beginning in January 2021 to distribute manpower and material resources to the COVID wards.

**Fig. 2:**
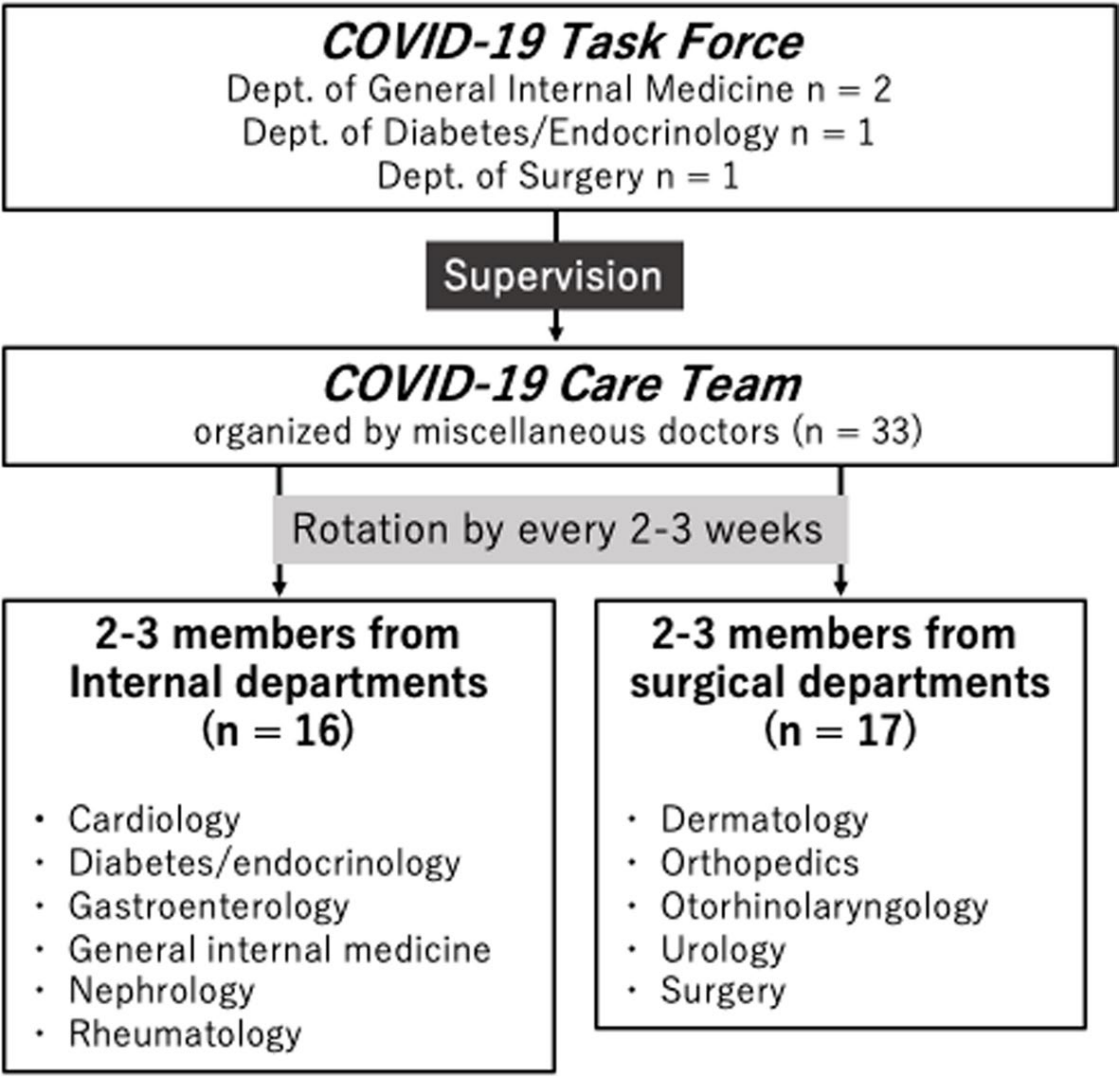
Organization chart of the COVID-19 duty. COVID-19: Coronavirus disease 2019

The Department of Pulmonology (*n* = 11) and the Department of General Internal Medicine (*n* = 4) had been in charge of hospitalized COVID-19 patients since April 2020. However, as the number of patients increased rapidly starting in mid-December 2020 and staff became overwhelmed, we decided to involve other departments in the inpatient treatment of COVID-19 patients. The COVID-19 duty was shared by doctors < 50 years of age on a 2- or 3-week rotation. The departments involved were cardiology, diabetes/endocrinology, gastroenterology, nephrology, rheumatology, dermatology, orthopedics, otorhinolaryngology, urology, and surgery (thoracic and abdominal). For convenience, we put these miscellaneous departments together and called them the COVID-19 Care Team. There were 4 doctors in the COVID-19 Care Team at a given time, which was subdivided into a team of 2 internists and a team of 2 surgeons (Fig. 2). This number increased to 6 doctors (3internists and 3 surgeons) during the 4th outbreak. Each participant treated approximately 5 to 6 patients during their duty period. The participants were not completely segregated from their non-COVID work, but they were reduced as much as possible. Entry into the red zone (area of viral contamination) was prohibited unless physical examination or a procedure requiring doctors, such as arterial blood sampling, were necessary. All participants had completed 2 vaccinations before their duty and received training for use of personal protective equipment (PPE).

The COVID-19 Care Team was supervised by the COVID-19 Task Force, which was comprised of 2 doctors with a strong expertise in infectious disease from the Department of General Internal Medicine and 2 members for support (Fig. 2). This task force was responsible for the development of a treatment algorithm and training of the participating doctors. The Task Force hosted daily evening meetings for both the Care Team and the Pulmonology Team to discuss management strategies and other aspects of treatment such as providing information. The Task Force was also available for individual consultation requiring immediate attention throughout the day, including the need to enter the red zone.

The objective of this study is to report our institutional effort to prevent task accumulation on specific healthcare workers and to investigate whether the inpatient COVID-19 treatment task can be shared by surgeons. The effects of this practice of involving surgeons in the treatment of hospitalized COVID-19 patients were evaluated by patient outcomes and potential physical/mental burden on the participating surgeons. We hope that our practice can provide a reference for task sharing among healthcare workers during a pandemic.

## Methods

### Characteristics of COVID-19 inpatients

Hospitalization of COVID-19 patients is decided upon request by the public health center of Kobe City. Requests are made when a patient has an SpO_2_ less than 94% at room air and for elderly patients with weakened activity. Advanced care planning (ACP) is confirmed with the patient and/or family members upon hospitalization. The most important aspect of a patient’s ACP is whether advanced respiratory management, such as mechanical ventilation and extracorporeal membrane oxygenation (ECMO), as well as cardiopulmonary resuscitation, is considered when necessary. To address the increasing number of patients, triage was performed based on age and health performance status. According to the criteria presented by the advanced care facility, patients older than 80 years of age were not eligible for advanced care, and upon discussion with the patient and his or her family, respiratory support was limited to oxygen masks or nasal high flow cannula (NHFC). Patients younger than 80 years old were eligible for transfer to an advanced care facility (ACF) when necessary, unless they had a poor performance status, had uncontrolled malignancies, or were incapable of making decisions due to dementia or other psychiatric conditions. Written informed consent was obtained from all patients prior to treatment. Severe patients eligible for intensive care were assigned to the Pulmonology Team, whereas elderly patients with severe disease and patients with mild to moderate severity were assigned to the COVID-19 Care Team.

### Study population and study design

**Fig. 3:**
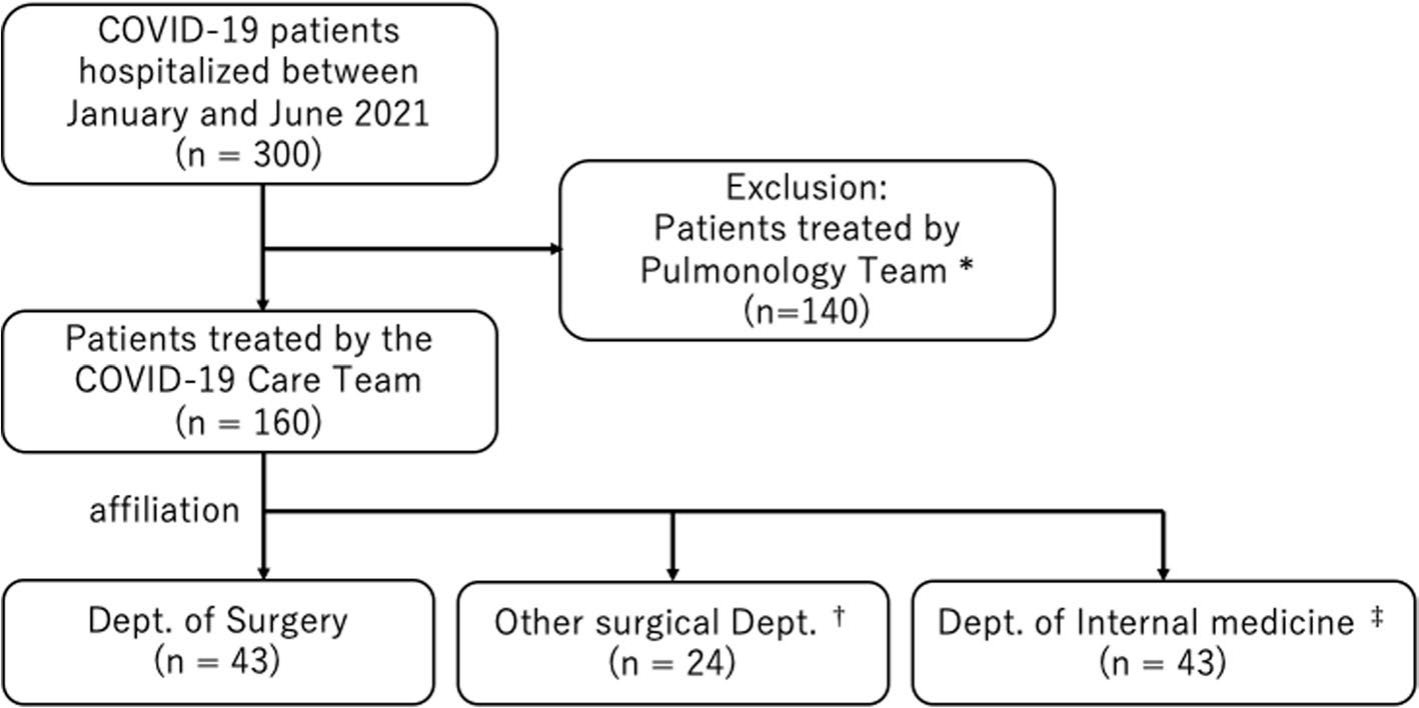
Study population. * Patients in the state of or at high risk of developing critical disease and are indicated for transfer to advanced care facility are assigned to the Pulmonologist team. † includes Dermatology, Orthopedics, Otorhinolaryngology, and Urology. ‡ includes Cardiology, Diabetes/endocrinology, Gastroenterology, General internal medicine, Nephrology, and Rheumatology

This is a single center retrospective observation study. The study population is shown in Fig. 3. There were 300 COVID-19 patients hospitalized in our institution from January to June 2021. Patients were either managed by the Pulmonology Team or the COVID-19 Care Team. The 140 patients with high risk for critical disease and eligible for intensive care were assigned to the Pulmonology Team, and were excluded since most of these patients were generally younger and were very likely to be transferred for intensive care due to their severity, which greatly affected patient outcomes. The remaining 160 patients managed by the COVID-19 Care Team were reviewed and divided into 3 groups depending on the affiliation of the attending physician: 1.) Department of Surgery, 2.) physicians in other surgical departments (dermatology, orthopedics, otorhinolaryngology, and urology), and 3.) internists (cardiology, diabetes/endocrinology, gastroenterology, general internal medicine, nephrology, and rheumatology).

First, the baseline characteristics and patient outcomes were compared between the 3 groups to validate our treatment protocol. The disease severity of the COVID-19 infection in this study complied with that of the United States National Institute of Health (NIH) [17]. In addition, the frequency of comorbidities that have been reported to be risk factors for critical disease were compared among the groups [18]. Second, to visualize the effect of the COVID-19 duty on the original tasks of surgeons, we compared the overtime working hours (per week per surgeon), the number of surgeries performed by each surgeon per week, and the frequency of surgical complications before, during, and after the duty. The stipulated work hours at our institution are from 9 A.M. to 5:30 P.M. on weekdays, and overtime work hours are any hours spent beyond those hours. The length of the preduty period and the postduty period depended on the length of each surgeon’s duty period: if the duty period was 2 weeks, we investigated the 2 weeks prior to and after the duty period, and if the duty period was 3 weeks, the 3 weeks prior to and after the duty period were investigated. The severity of the complication was defined in accordance with the Clavien-Dindo classification (CD) [19], and the frequency was calculated by counting the number of complications with CD grade III or higher and dividing it by the total number of elective operations during each period. Third, to investigate matters that are difficult to objectively evaluate, a survey was conducted on the participating surgeons. Questions about changes in their personal lives, the presence of physical and mental burdens, and the motivation for COVID-19 duty were answered by all 8 participants. The questions in the survey were answered yes or no, and respondents could give any specific details with the answer given.

All study protocols were approved by the Institutional Ethics Board (approval no. 21-017), and all procedures were conducted in accordance with the Declaration of Helsinki 1996.

### Task-shifting of surgeons

From the Department of Surgery, 8 members out of 13 were assigned the COVID-19 duty, of which 4 were residents (≤3 years of surgeon experience). Four surgeons had a 2-week duty, and the other 4 members had a 3-week duty. One member was part of the COVID-19 Task Force and was exempted. The remaining 4 members were excluded because of their age (≥50 years old). During the duty period, surgeons were asked to prioritize their COVID-19 duties, and although they were not completely excluded from their surgical duties, they were intentionally reduced.

### Treatment protocol of COVID-19 patients

**Fig. 4:**
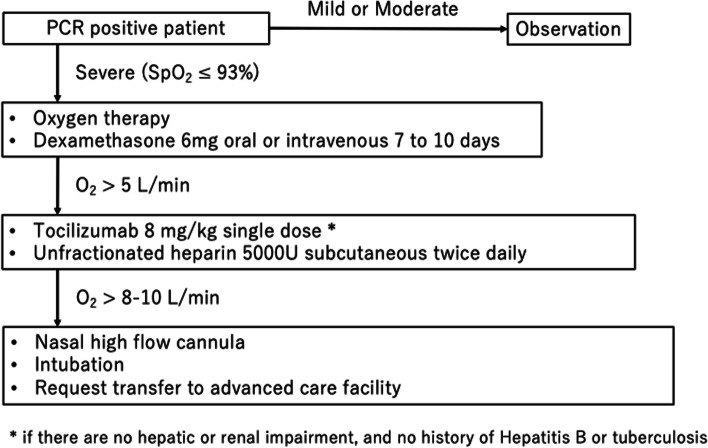
COVID-19 treatment algorithm. COVID-19: Coronavirus disease 2019. PCR: Polymerase chain reaction

The treatment protocol in our facility is based on the treatment/management guidelines set by the Ministry of Health, Labour and Welfare of Japan and in agreement with those of the National Institute of Health (NIH) and the World Health Organization (WHO) (Fig. 4) [17, 18]. Patients underwent a routine blood and chest X-ray examination upon hospitalization. Patients with sufficient oxygen saturation in their blood (SpO_2_ ≧94%) were observed for disease progression. When SpO_2_ was 93% or lower, oxygen therapy was started, along with a 10-day administration of 6 mg dexamethasone. When the oxygen dose exceeded 5 Liters/minute (L/min), a single 8 mg/kg dose of tocilizumab was administered if there were no hepatic or renal impairments or a history of hepatitis B or tuberculosis. Multipurpose oxygen masks were switched to a high-flow nasal cannula (HFNC) if tolerable. Since April 2021, intubation of up to 2 patients was performed at our facility due to the increase in critically ill patients, and patients in need of intubation were passed on to a pulmonologist. Indications for transfer to an advanced care facility varied throughout the study period. In January 2021, a transfer was requested if the oxygen dose reached 4 L/min, but by April, transfer requests could only be made for patients who were intubated or very close to being intubated (FiO_2_ > 80% on HFNC). The requirements for discharge and rehabilitation transfer were as follows: 1.) 10 days from onset and 2.) 3 days after resolution of fever. Medical records were recorded based on a template provided by the COVID-19 Task Force.

### Statistical analysis

Continuous variables are presented as medians with ranges or interquartile ranges (IQRs) as appropriate. Categorical variables are presented as numbers and percentages. Comparisons were performed using the Kruskal-Wallis test for continuous variables and the χ^2^ test or Fisher exact test for categorical variables as appropriate. A *P* value of <.05 was considered to indicate statistical significance. EZR (Saitama Medical Center, Jichi Medical University, Saitama, Japan), a graphical user interface for R (The R Foundation for Statistical Computing, Vienna, Austria), was used for all statistical analyses [20].

## Results

### Baseline characteristics and outcome of COVID-19 patients

The patient characteristics are summarized in Table 1. There were 75 men (46.9%) and 85 women (53.1%), and the median age was 82.5 years old (range, 16–103), of which 99 patients (61.8%) were 80 years-old or older. Disease severity was as follows: 11.2% for mild, 8.7% for moderate, 44.3% for severe, and 26.8% for critically ill. In terms of outcomes, 36.9% were discharged home, 28.1% were transferred for rehabilitation, 3.8% were transferred to ACF, and 31.2% died. Finally, patients were assigned to each group as follows: 43 to the Department of Surgery, 24 to other surgical departments, and 93 to internist departments. No significant differences were observed in age, sex, or comorbidities. The frequency of disease severity and the presence of co-infections were also similar among the groups. The median length of hospital stay (14 (1-53) vs 14 (3-41) vs 15 (1-69) days; *P* = .937) and the outcomes (Discharge: 34.9% vs 45.8% vs 35.5%; *P* = .626; Rehab transfer: 30.2% vs 25.0% vs 28.0%; *P* = .910; ACF transfer: 0% vs 1% vs 5.4%; *P* = .287; Death: 34.9% vs 25.0% vs 31.2%; *P* = .722) were similar among the groups.

**Table 1:** Baseline characteristics and outcome of COVID-19 patients within the COVID-19 care team

Variables	Affiliation of treating doctor			*P* value
	Surgeon (*n* = 43)	Other surgeon^a^ (*n* = 24)	Internist (*n* = 93)	
Age, years	82 (62 - 92)	87 (16 - 103)	82 (49 -101)	.405
Age ≥ 80 years	27 (62.8%)	17 (70.8%)	55 (59.1%)	.565
Sex, female	21 (48.8%)	18 (75%)	46 (49.5%)	.065
Comorbidities				
Hypertention	24 (55.8%)	11 (45.8%)	56 (60.2%)	.434
Diabetes mellitus	14 (32.6%)	7 (29.2%)	28 (30.1%)	.970
Heart disease	8 (18.6%)	6 (25.0%)	21 (22.6%)	.772
Cerebrovascular disease	5 (11.6%)	3 (12.5%)	16 (17.2%)	.743
Chronic pulmonary disease	6 (14.0%)	2 (8.3%)	8 (8.6%)	.569
Chronic kidney disease	2 (4.7%)	3 (12.5%)	5 (5.4%)	.391
Active malignancy	3 (7.0%)	1 (4.2%)	5 (5.4%)	.890
Body mass index ≥30 kg/m^2^	0 (0%)	1 (4.2%)	3 (3.2%)	.473
Active immunosuppresion	1 (2.3%)	0 (0%)	4 (4.3%)	.837
Dementia	13 (30.2%)	13 (54.2%)	29 (31.2%)	.098
Severity				.376
Mild	6 (14.0%)	2 (8.3%)	10 (10.7%)	
Moderate	3 (7.0%)	5 (20.8%)	6 (6.5%)	
Severe	16 (37.2%)	11 (45.8%)	44 (47.3%)	
Critical	18 (41.8%)	6 (25.0%)	33 (35.5%)	
Coinfection	8 (18.6%)	7 (29.2%)	28 (30.1%)	.352
Hospital stay, days	14 (1 - 53)	14 (3 - 41)	15 (1 - 69)	.937
Outcomes				
Discharge to home	15 (34.9%)	11 (45.8%)	33 (35.5%)	.626
Transfer for rehabilitation	13 (30.2%)	6 (25.0%)	26 (28.0%)	.910
Transfer to advanced care facility	0 (0%)	1 (4.2%)	5 (5.4%)	.287
Death	15 (34.9%)	6 (25.0%)	29 (31.2%)	.722

Continuous variables are presented as medians (ranges). Categorical variables are presented as numbers and percentages

^a^Urology, Dermatology, Otorhinolaryngocology, Orthopedics

### Change in overtime working hours of surgeons

**Fig. 5:**
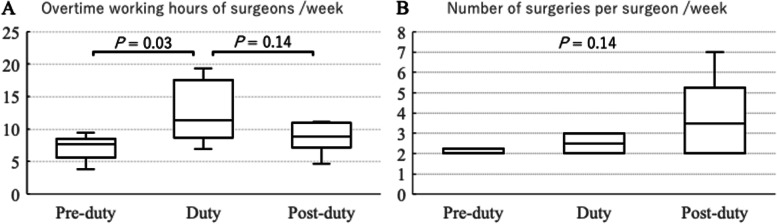
The comparisons of factors associated with surgeon’s tasks. **A** Box and whisker plot of overtime working hours for each surgeon per week. The increase from the preduty period to the duty period was significant (*P* = 0.03). **B** Box and whisker plot of the number of surgeries performed by each surgeon per week (*P* = 0.14). Continuous variables were compared using the Kruskal-Wallis test with significance set at *P* < 0.05

The overtime working hours per week of each surgeon before, during and after the COVID-19 duty (between the 3 different periods) were compared (Fig. 5A). The median overtime hours (11.44 hours/week; range: 6.93–19.33) during the duty period were longer than that during the preduty period (7.75 hours/week; range: 3.83–9.50, *P =* 0.03). The median hours during the duty period were longer than the postduty period, but the difference was not significant (8.88 hours/week; range: 4.67–11.17, *P =* 0.1).

### The number of surgeries and outcomes of surgical patients

The number of surgeries performed by each surgeon per week was compared between the 3 different periods (Fig. 5B). The median number was 2.0 patients per surgeon per week (range: 1–4) before the duty, 2.5 (range: 2–5) during the duty, and 3.5 (range: 2–7) after the duty, with no significant difference (*P* = .137). The frequency of postoperative complications with CD grade ≧III was comparable among the periods (0 (0-0) vs 0 (0-1) vs 0 (0-1) patients/week, *P* = .460).

### Questionnaire survey results

The results of the questionnaire survey answered by 8 surgeons are summarized in Table 2. One participant lived separately from his family, and 3 made changes to their personal lives due to fear of transmission. Specifics included immediate showering upon returning home or sleeping in separate bedrooms to avoid spreading the virus at home. Half of the participants answered that their surgical duty was affected by their COVID-19 duty by being less involved in surgeries.

**Table 2:** Questionnaire survey results. The percentage and fraction of the participants who answered yes to each question is shown

Questions	Answers, yes	Specifics
Did you live separately from your housemate during your period of duty?	12.5% (1/8)	
Any changes in your personal life?	37.5% (3/8)	• Immediate showering upon return
		• Separate bedrooms
		• Unpleasant treatment by family member
Did you have to go into the red zone?	75.0% (6/8)	
Any effect on your surgery duty?	50.0% (4/8)	• Less involvement in operations
Any mental burden?	87.5% (7/8)	• Anxiety about getting infected
		• Providing informed consent to families of unrescuable patients was emotionally difficult
		• Although there was a treatment protocol, there was mental stress from taking part in treating an unfamiliar condition
Any physical burden?	37.5% (3/8)	• Having to attend the mandatory meetings everyday was a burden
		• Having to use the phone for communication with patients and families
		• Examining patients with personal protective equipment
Will to participate?	25.0% (2/8)	

In terms of burden, 75% of the participants had to enter the red zone (area with COVID-19 contamination) at least once during their duty with full PPE (personal protective equipment). A majority (87.5%) suffered from mental burden, which mainly involved fear of transmission and concerns related to treating patients with a disease that they were not experienced with. In addition to the mental burden, some reported physical burdens (37.5%) from mandatory daily meetings and communicating with patients through phone calls. In contrast, only a quarter of the participants showed a positive will to participate in the COVID-19 duty.

## Discussion

With facilities adjusting to the rapid spread of SARS-CoV2, there were precedents of surgeons being redeployed to treat non-surgical COVID-19 patients [7–14]. However, not much light has been shed on the system that supports surgeon involvement without detrimental effects on patient outcome and surgeon welfare. Our practice proved that physicians with different fields of expertise can safely share the task of inpatient care of COVID-19 patients with treatment standardization and support by a supervisory unit. As a result, we were able to prevent excessive workload on our pulmonologists by distributing 53.3% (160/300) of the patients to miscellaneous physicians. However, from the standpoint of surgeons, there were both positive and negative aspects that raised several issues for discussion.

First, this “rotation system” was developed to care for as many patients as possible in an efficient manner with limited resources. A standardized treatment protocol and sufficient support by a supervising group was essential in involving doctors with not much training in the management of COVID-19 patients, let alone, a respiratory infection. Our treatment protocol was designed to be clear and concise in the indication and use of treatment agents. In addition to the standardization of treatment, the universal format of the medical records allowed smooth exchange of information among the participating members. However, with over half of the COVID-19 Care Team patients being older than 80 years old and having problems besides COVID-19 treatment, a standardized protocol was not enough to ensure the quality of management. The evening meetings and availability of the COVID-19 Task Force to consultation throughout the day have played a critical role in addressing this problem. Since old age is associated with higher severity and higher mortality [21], the mortality of our cohort was unfortunately high. Nevertheless, the results of our study have shown that the patients of our cohort have received equitable care regardless of the background of the treating doctor. As a note, the proportion of residents in each subgroup was similar (37.5% for the department of surgery vs 55.6% for other surgical departments vs 47.1% for internists, *P* = .903), and thus the expertise of individual experience did not vary among the subgroups.

While involving surgeons in COVID-19 treatment was necessary, it was important to ensure that their self-esteem and satisfaction were preserved [22]. To maintain this balance, participating surgeons were not completely segregated from non-COVID activities. Although team segregation is important in preventing disruption of surgical services [10, 11], there was a concern for the negative effect on such segregation on the training of our early-career surgeons, since decreased operative experience is reported to have a strong influence on them [5, 23, 24]. As a result, each surgeon was able to perform at least 2 surgery per week as the operator (Fig. 5), and although some felt a reduction in their surgical activity, there were no voices of anxiety related to reduced training experience. In addition to the training purposes, complete segregation was avoided because it was difficult to fulfill both the operational, outpatient, and emergency demands, despite the reduced hospital activity during the study period. This was the case with every other department, and so we put effort into minimizing the risk of viral transmission among and beyond the Care Team participants by setting a strict limitation to red zone entry and providing adequate training and supply of PPE use. Fortunately, there was no reported transmission among the participants, but considering what has been reported in past literature, the segregation of teams must be considered if the availability of manpower allows for it. Therefore, further investigation is necessary to reach the optimal balance between maintaining surgeons’ self-esteem and having them fulfill their responsibilities as physicians.

Confronting the burden associated with task shifting is also important. There was a significant increase in overtime working hours despite less involvement in surgical tasks, which is thought to be the result of irregular emergency admission, late-time meetings, and weekend duties, suggesting that the COVID-19 duty was taking a physical toll. In our data, 87.5% of the participants reported mental burden from the COVID-19 duty, compared to 37.5% reporting physical burden. A study on the effect of the redeployment on surgical trainees reported that 50% of the participants felt negative effects on their mental health [14]. This indicates that such obligations can be detrimental to the mental health of the participating doctors. The main concerns of the participating surgeons involved the management of an unexperienced disease and the stress from informing families about the deteriorating course of patients. A similar finding was reported through a survey on orthopedic trainees who experienced redeployment during the pandemic era [12]. To alleviate anxiety in patient management, the COVID-19 Task Force put great effort into listening and providing feedback to the participants.

### Limitations

Although our practice was unprecedented, there were several limitations. First, this is a practice at a single institution, and our system may not be adaptable by all facilities since available resources and care systems vary among nations and institutions. In addition, the success and problems of surgeon involvement may not have been fully revealed without comparing our results to other facilities that have managed COVID-19 patients without involving surgeons. However, comparison with such facilities could not be made since information on whether other facilities involved surgeons was absent, and thus such data was unavailable. Yet, we were able to find out that this task-sharing system did at least reduce the workload on the Department of Pulmonology by assigning 160 patients to physicians from other departments within our institution. Second, with the introduction of multiple new agents in addition to vaccination, treatment strategies have become more complex and difficult to develop protocols for [17, 25, 26]. The treatment protocol needs repeated updates in correspondence with the changing situation and the facility’s capacity. Finally, our data of overtime working hours and complications do not necessarily reflect the true influence of the COVID-19 task on participating surgeons, since surgical cases were significantly reduced during the COVID-19 crisis. Nonetheless, with the pandemic hardly coming to an end and new variants emerging, it is important to analyze these data objectively and identify problems for refinement. Our findings can be a valuable lesson for the next pandemic.

## Conclusion

In conclusion, surgeons can successfully share the task of inpatient COVID-19 treatment with the implementation of a treatment algorithm and a supporting task force. However, as such duty could negatively affect the surgeons’ physical and mental wellbeing, further effort is needed to seek the optimal balance of fulfilling individual needs as well as the needs of the institution and community.

## Data Availability

The data from the present study are available upon reasonable request. Please contact the first author for permission of usage of the original data. (Email: ymatsui@kcho.jp).
